# Pre- and Posttherapeutic Staging of Laryngeal Carcinoma Involving Anterior Commissure: Review of 127 Cases

**DOI:** 10.5402/2012/363148

**Published:** 2012-09-10

**Authors:** Marc Foucher, Raphaëlle Barnoud, Guillaume Buiret, Jean-Christian Pignat, Marc Poupart

**Affiliations:** ^1^CHU La Croix-Rousse, Otolaryngology and Head and Neck Surgery Department, Claude Bernard University Lyon 1, 69317 Lyon, France; ^2^CHU La Croix-Rousse, Biology and Pathology Center, Claude Bernard University Lyon 1, 69317 Lyon, France

## Abstract

*Background*. The objective of this study is to assess the accuracy of pre- and posttherapeutic staging of endolaryngeal cancer involving anterior commissure. *Materials and Methods*. 127 patients were included in this retrospective study, and laryngectomy (partial or radical) was achieved in all of them. Initial radioclinical evaluation (cT) was performed (endoscopy-CT scan) and compared with postoperative histopathological findings. *Results*. 24,6% of cT2 and 33,3% of cT3 laryngeal tumors were reclassified pT4 after the histopathological examination. *Conclusion*. pre-therapeutic staging (combining endoscopy-CT scan) of endolaryngeal cancer involving anterior commissure is inadequate and sometimes underestimates thyroid cartilaginous invasion. Nethertheless, a precise diagnostic assessment by surgery with postoperative histological findings is possible. Cartilage and/or paraglottic structures are involved, or not, on the laryngectomy specimen exam. So surgery should always be discussed in first line in transdisciplinary meeting for endolaryngeal cancer management.

## 1. Introduction

Laryngeal cancers represent about 30% of head and neck cancers with a high incidence for the glottic location (from 25 to 85%) [1, 2]. 

There is currently no recommendation from the UICC (International Union Against Cancer) for pre-therapeutic assessment (endoscopy, computed tomography, and magnetic resonance imaging) specifying in particular the need to involve them.

Now the evaluation of the cartilaginous extension, especially thyroid, is an important element in the pre-therapeutic assessment of the endolaryngeal cancer. In case of infiltration the tumor is classified cT4a, and the patient is amenable to an aggressive therapy given the high risk of local recurrence and low radiosensitivity. The same applies to the infiltration of paraglottic areas.

The purpose of this study is to evaluate, by comparing, the existing correlation between preoperative radiological classification of endolaryngeal tumors involving the anterior commissure (endoscopy, CT scan) and postoperative classification (pT pathology).

## 2. Materials and Methods

### 2.1. Patients

This is a single-center retrospective study (1998–2005) conducted at the Croix-Rousse hospital on 127 patients with endolarynx cancer involving the anterior commissure. Men are in majority (122 cases). All these patients were surgically treated and 32 of them with a total laryngectomy, the 95 other patients with a partial laryngectomy. Squamous-cell carcinoma represented the main pathology (124 patients) with a case of adenosquamous carcinoma, a case of pseudosarcomatous carcinoma, and a last case of verrucous carcinoma.

### 2.2. Clinical, Endoscopic, Radiological and Histological Assessment

All patients underwent a nasofiberscopy (including a verification of chordal mobility) followed by panendoscopy using optical at 0° and 30°. Radiological examinations were performed in seven different centers in 2 mm maximum sections, in all cases after injections of contrast. The tumor extension should be clarified to the following areas: sub glottis, paraglottic area, preepiglottic area, and cartilages (thyroid, cricoid). 

### 2.3. Tumors Classification

Tumors were classified according to the UICC 2002 classification [6]. 

## 3. Results

The patients were 58 years old in average, 93% of them were smokers, and more than half had a regular alcohol consumption.

Table 1 summarizes the comparison of cT and pT stages for all tumors in the study.

Only 76% of tumors have been classified correctly with the endoscopy and CT scan combination. It is more likely to correctly classify tumors with a limited size since only 64% of T3 and T4 tumors were correctly classified preoperatively. 

24.6% of CT2 and 33.3% of CT3 tumors are actually reclassified pT4 after histopathological examination. This shows that the cartilage invasion is frequently under-estimated by the radiological assessment with endoscopy and cervical CT scan. 

This underestimation of the cartilage infiltration is more common than on the assessment of the subglottis, paraglottic areasc and preepiglottic extension. Only 2% of cT2 tumors were reclassified pT3, and 5% of the cT3 tumors were reclassified pT2 after pathological examination of the specimen. 

## 4. Discussion

After the publication of Hoffman et al. [7] in a retrospective study showing a decrease in the survival rates of laryngeal cancer in the United States, the terms of support, especially for the advanced stages, are currently controversial. 

If no conclusion of this study can be drawn in a formal and dogmatic manner, the proliferation of treatment options (radical surgery, partial surgery, transoral surgery, and laryngeal preservation protocols) requires more accurate and right pretreatment staging. One of the major means of all these studies, randomized or not, is the selection mean with the inclusion of patients with faulty pretreatment radiological staging. 

The underestimation of the thyroid cartilage invasion by cervical CT is found in many studies with variable rates. In a prospective study related to the cartilage invasion, realized on 40 patients, Zbären et al. found a 67% sensitivity and a 87% specificity of CT [8]. 

When studies involve all endolaryngeal tumors, it appears that the anterior commissure of the underestimation is greatest. Thus, on Nakayama and Brandenburg's cases, 50% of T3 tumors were subclassified by breach of a micro-cartilage invasion [9]. 90% of this situation involved tumors of the anterior commissure. 

The special case of the thyroid cartilage invasion via the anterior commissure was also clearly demonstrated. Barbosa et al. then found a 25% overall underestimation of the cT stage of the anterior commissure tumors by the combination CT/endoscopy [5]. However, reports show a stronger underestimation for smaller tumors classified as T1 and T2 (respectively 50% and 62% of correct estimation). 

Agada et al. showed that the redefinition of radiological criteria allowed the increase in accuracy of radiological conclusions in two successive audit cycles by increasing the accuracy rate of 45% to 71% [3]. This also shows that our balance sheet allows us to achieve correct figures and that theses are not related to poor quality of the examinations. 

Table 2 summarizes the various studies on the reclassification of endolaryngeal tumors after postoperative pathologic examination (initial radiological staging CT/endoscopy). 

Some scan signs to look for can increase the relevance of this paper. For the thyroid cartilage, specificity (ability to correctly identify individuals who are not affected by the disease) would be about 93% for erosion or lysis. It would amount to 95% in case of an extracartilaginous extension. The combination of these signs causes a high sensitivity (ability to detect cases of a disease) and reinforces the negative predictive value (probability of being healthy if negative) [10]. 

Zbären et al. also studied retrospectively the reclassification of the endolaryngeal recurrent lesions [11]. In these cases, it appears that the combination CT/endoscopy is even less accurate to correct a TNM stage assessment. They report indeed a 48% sensitivity on the study of the thyroid cartilage invasion and 47% for the cricoid invasion. 

Our study did not use any MRI as an examination to detect the cartilage infiltration. With a 89–94% sensitivity and a 74–88% specificity, it is for sure an interesting radiological exploration. Its negative predictive value (NPV) is around 94–96%, but its positive predictive value (PPV) reaches only about 71% (probability of getting sick if positive). They are then many false positives with a significant risk of inadequate treatment by overestimation [12–15]. 

This difficult pretreatment evaluation of the cartilaginous extension of endolaryngeal tumors leads us to suggest a surgical approach with resection of exposed cartilaginous portions as soon as the anterior commissure and the anterior portion of the subglottis are getting invaded. 

## 5. Conclusion

Our study shows that radiological pretreatment classification (cT) of laryngeal cancer involving the anterior commissure is often inaccurate when compared with postoperative pathology (pT). The MRI appears to offer a more effective accuracy but still below the pT. 

This finding should lead to a transdisciplinary consideration from the moment the treatment choice is not directed towards a first surgical resection.

## Figures and Tables

**Table 1:** Summary of stages cT/pT for cancers reaching the anterior commissure.

				pT			Total
			1	2	3	4
	1	%/No. of patients	66,7 (4)	16,7 (1)	—	16,7 (1)	100,0 (6)
cT	2	%/No. of patients	—	73,8 (45)	1,6 (1)	**24,6 (15) **	100,0 (61)
	3	%/No. of patients	—	4,8 (1)	61,9 (13)	**33,3 (7) **	100,0 (21)
	4	%/No. of patients	—	7,7 (3)	5,1 (2)	87,2 (34)	100,0 (39)

Total		%/No. of patients	3,1 (4)	39,4 (50)	12,6 (16)	44,9 (57)	100,0 (127)

**Table 2:** Summary of various studies on the reclassification of laryngeal cancers after surgery. Endolarynx: no precision on the tumor site, CA: anterior commissure.

Study	Type of study	No. of patients	Tumor location	Reclassification rate
Remontet et al. [1]	Prospective	40	Endolarynx	20%
Agada et al. [3]	Prospective 1st cycle of an audit	38	Endolarynx	55%
	Prospective 2nd cycle of an audit	38	Endolarynx	29%
Zbären et al. [4]	Retrospective	42	Recidivism	36%
Barbosa et al. [5]	Prospective	52	CA	25%
Our study	Retrospective	127	CA	24,4%
